# Acute myeloid leukaemia presenting as acute liver failure—a case report and literature review

**DOI:** 10.3332/ecancer.2019.960

**Published:** 2019-09-09

**Authors:** Kai Sun, Ryan J Reynolds, Tiffany G Sheu, Jessica A Tomsula, Lara Colton, Lawrence Rice

**Affiliations:** 1 Department of Medicine, Houston Methodist Hospital, Houston, TX 77030, USA; 2 Department of Pathology and Genomic Medicine, Houston Methodist Hospital, Houston, TX 77030, USA; 3 Weill Cornell Medical College, Houston, TX 77030, USA

**Keywords:** acute myeloid leukaemia, acute liver failure, obstructive jaundice

## Abstract

A 75-year-old woman presented with rapidly progressive fatigue, abdominal pain and jaundice. Physical examination revealed tender abdomen and splenomegaly. Magnetic resonance cholangiogram showed marked hepatomegaly, splenomegaly and scattered nodules or masses in the liver and spleen. The patient expired from multiorgan failure. Autopsy revealed infiltration of the liver, spleen and bone marrow by acute myeloid leukaemia.

## Introduction

In patients presenting with acute liver failure (ALF), acute myeloblastic leukaemia (AML) is extraordinarily rare and is associated with very high mortality. We cared for a patient who presented with acute hepatic failure and was diagnosed with AML on autopsy (albeit suspected immediately pre-mortem). We review the literature to gain insights into this problem and how outcomes might be improved.

## Case presentation

A 75-year-old woman presented to another hospital with fatigue, right upper quadrant abdominal pain and jaundice, was evolving over several days. She denied fever, sick contacts, recent infectious symptoms, recent travel or new medications. Past medical history was remarkable only for moderate Parkinson’s disease. Medications were aspirin, calcium-vitamin D3 and rasagiline. She was afebrile and jaundiced. Haemoglobin was 8.9 g/dL; white blood cells 6.35 k/uL with 63% neutrophils, 21% lymphocytes and 13% monocytes; platelets 30 k/uL; alkaline phosphatase 92 U/L; aspartate aminotransferase (AST) 62 U/L; alanine aminotransferase (ALT) 104 U/L and total bilirubin 4.5 with 2.4 mg/dL direct fraction. Tests for Epstein–Barr virus, cytomegalovirus, hepatitis and human immunodeficiency viruses were negative. Autoimmune workup was negative, including anti-nuclear antibodies, anti-mitochondrial antibody, anti-Smith antibody and liver-kidney microsomal antibody. Magnetic resonance cholangiogram showed marked hepatomegaly, moderate splenomegaly and nodular areas of decreased T2-weighted signal within the liver and spleen parenchyma. She clinically deteriorated over a few days.

**Figure 1.**
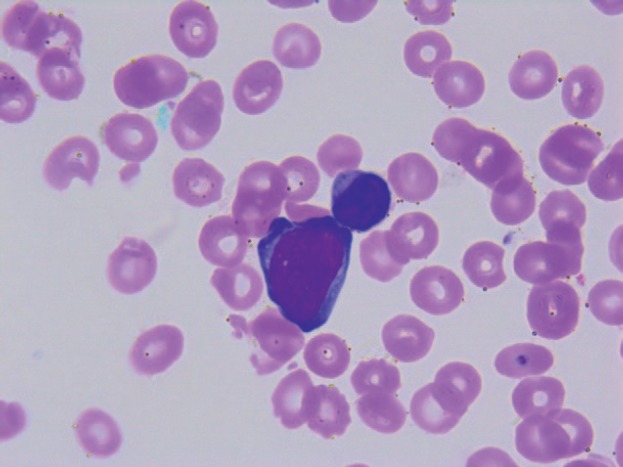
Peripheral smear.

On transfer to Houston Methodist Hospital, she appeared acutely ill and moderately confused. The abdomen was distended, tense and tender in the right upper quadrant; the spleen was palpably enlarged. The white blood cell count remained 6.29 k/uL with 75% neutrophils, 18% lymphocytes and 5% monocytes; haemoglobin 7.9g/dL; platelets 9 k/uL; alkaline phosphatase 92 U/L, AST 34 U/L and ALT 79 U/L; total bilirubin 16.4 mg/dL; direct bilirubin more than 10 mg/dL; lactate dehydrogenase (LDH) 123 U/L and prothrombin time 21.2 s, internationalised ratio 1.8, partial thromboplastin time 36, D-dimer 3.41 and fibrinogen 159 mg/dL. Florid encephalopathy rapidly ensued, as AST increased to 1,669, ALT to 518, total bilirubin to 19.8, along with acute renal failure, worsening coagulopathy and severe metabolic acidosis. A haematology consultant noted rare large blast cells on the peripheral smear (3 of 200 cells, Figure 1); peripheral blood flow cytometry did not detect these. The patient expired within few days of initial presentation before planned liver and bone marrow biopsies could be performed.

**Figure 2.**
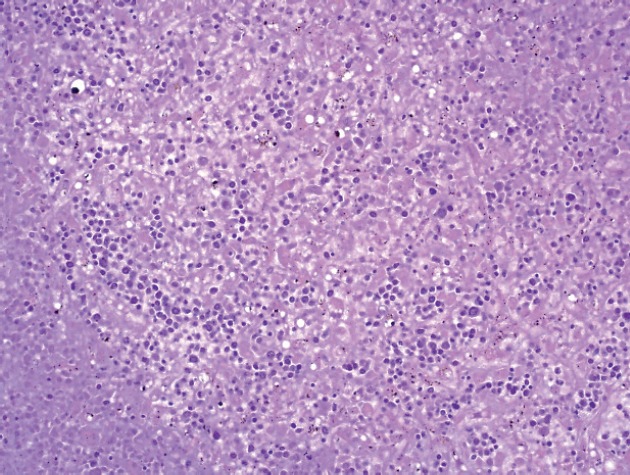
Microscopic examination of the liver.

**Figure 3.**
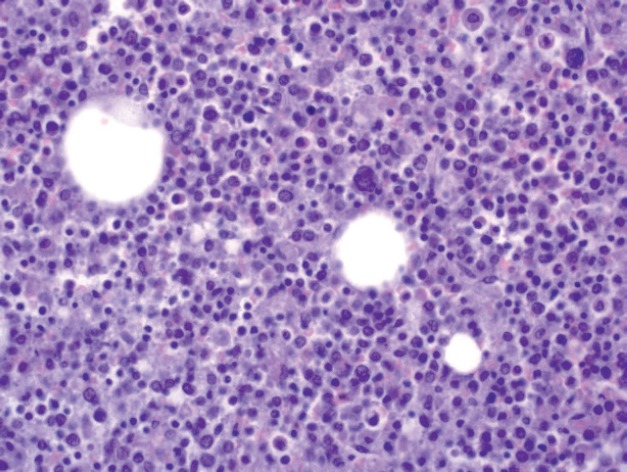
Microscopic examination of bone marrow.

A limited autopsy of the liver, spleen and bone marrow revealed gross hepatosplenomegaly with diffusely mottled hepatic parenchyma and variably soft and firm red-black nodular spleen. Microscopic examination of the liver and spleen found effacement of the normal architecture by a diffuse neoplastic infiltrate (Figure 2). Residual hepatocytes were necrotic. The neoplastic cells had scant cytoplasm, immature nuclear chromatin and moderate nuclear pleomorphism. Bone marrow (Figure 3) was hypercellular (80%) with expansion by neoplastic cells. While few residual erythroid and megakaryocytic elements were identifiable, there were no maturing myeloid precursors. Immunohistochemical stains confirmed the myeloid lineage of the neoplastic cells (strong positive myeloperoxidase) which also expressed monocytic (lysozyme) and erythroid (E-cadherin) markers. Subclassification of the AML was limited by cellular degeneration, so the process was classified as acute myeloid leukaemia, not otherwise specified.

## Discussion

ALF is a life-threatening disease with high mortality rate [1]. The most common causes of ALF are drugs (acetaminophen is most common [2]) and viral infections [3]. Neoplastic infiltration as a cause of ALF is rare. In a large single centre study performed by Rowbotham *et al.* [4], only 18 out of 4,020 patients (0.44%) had their ALF attributable to neoplastic infiltration. Rich *et al.* [5] found only 27 of 1,910 such cases (1.4%). The most common malignancies in those studies were lymphoma (41%–79%) and metastatic breast cancer (30%), whereas only two patients had ALF from acute leukaemia. AML as a cause of ALF is reported far less commonly than acute lymphoblastic leukaemia (ALL) in children [6–8] and in adults [9–13] but has been described in several case reports.

The first report of ALF due to AML was by Buchler and Cline [14] who described a young female with initially vaginal lymphoma; no details of the AML were provided in the case report. Seven subsequent cases of AML presenting as obstructive jaundice or ALF without distinguishable obstructive masses have been reported [12, 15–20] (see Table 1). All patients had abnormal blood counts that were reflected as anaemia and/or thrombocytopenia and/or leukocytosis. Blasts were seen on peripheral smears in five of seven patients. Five of seven patients had liver and/or spleen enlargement detected on physical examination, abdominal imaging or autopsy.

In those cases, the ALF was manifest as cholestatic, hepatocellular or a mixed pattern, but hepatic sinusoidal infiltration was universally seen on liver biopsies. The patients who presented with very high bilirubin also had hepatocyte necrosis. We suspect that the liver damage first starts with hepatic sinusoidal infiltration causing ischaemia, then progresses to liver failure when the damage leads to liver tissue necrosis. Several cases [21–23] have described granulocytic sarcoma as the cause of obstructive jaundice which could be another mechanism of ALF in acute myeloid leukaemia. Even though our patient had mass-like lesions on imaging, the histologic examination revealed a diffuse involvement of the hepatic and splenic tissue.

The diagnosis of ALF due to AML is generally made by liver biopsy or by autopsy. While AML can present without circulating blasts, blasts on peripheral smear would typically point to the diagnosis of AML. In our case, none of the laboratory blood counts reported the presence of blasts, and even when rare peripheral blasts were noted by a haematology consultant, these were not detected by flow cytometry. Nevertheless, even if the diagnosis could have been made in a more timely fashion, this condition still engenders very high mortality. In the two previously mentioned case studies [4, 5], mortality rate was 100%. In case reports, only two out of eight patients survived after chemotherapy.

## Conclusion

Even though AML as a cause of obstructive jaundice or ALF is rare, it should be considered as differential if patients have abnormal complete blood count (CBC) and hepatic or splenic enlargement. Peripheral smears should be done first in a timely manner if abnormal CBC is present. If unexplained abnormalities are present on peripheral smears in the setting of abnormal CBC and hepatomegaly or splenomegaly, bone marrow or liver biopsy should not be delayed to rapidly establish a diagnosis and timely initiate chemotherapy which would afford the patient some reasonable chance of remission and survival.

## Funding

No funding was received for this case report.

## Conflicts of interest

The authors declare that they have no conflicts of interest.

## Figures and Tables

**Table 1.** Summary of case reports of AML presenting as obstructive jaundice or ALF.

Authors	Age/ Gender	CBC	Liver function	LDH	Smear	Bone marrow	Liver biopsy	Imaging	Outcome
Goor *et al.* [15]	36/M Hepatomegaly on exam	WBC 4.6 40% blasts Hgb 12.5 Plt 176	Tbil 3.5 AST 350 ALT 360	–	–	AML	Not done	US: dilatation of intrahepatic bile duct, normal size CBD and thickening of the gallbladder wall	CR after chemo
Wandroo *et al.* [16]	40/M	WBC 23.5 (N 26.7% L 15.7%, Mono 52.8%, E 2%, blasts 0.8%) Hgb 11.4 Plt 75	Tbil 4.68 AST 142	–	Dysplastic monocytes with occasional monoblasts	Increased number of myeloid and monocytic cells with few blasts	Diffuse sinusoidal infiltration by a pleomorphic population of cells, mostly consisting of neutrophil and monocytes, with some immature blasts	CT: hepatosplenomegaly; altered echogenicity of the liver but normal biliary ducts	CR after chemo
Sobotka *et al.* [20]	66/F	WBC 3.1 Hgb 8.6 Plt 18	Tbil 3.4 Dbil 1.7 AST 38 ALT 26	–	–	Dry tap	Diffuse infiltration of the liver with left-shifted erythroid precursors and hepatocellular cholestasis	MRI: coarse echotexture of the liver, normal bile ducts	Death
Rajesh *et al.* [17]	32/M	WBC 10.2 (N 65%, L 13%, Mono 20%, E 2%, B 1%) Hgb 11.9 Plt 289	Tbil 24.9 Dbil 14.7 AST 52 ALT 60	–	Blasts	75% peroxidase-positive myeloid blasts with large number of eosinophils	–	CT: hepatomegaly, dilatation of intrahepatic ducts and CBD	Death
Mathews *et al.* [18]	66/F	WBC 12.4 Plt 63	AST 49 ALT 73 No cholestasis		Few circulating blasts	Confirmed AML	Hepatic sinusoidal infiltration with AML	MRI: an 11 mm T2 hyperintense lesion in the posterior dome of the liver	CR after induction chemo but death after relapse
Anderson *et al.* [12]	30/F	WBC 114 Hgb 13.6 Plt 70	Tbil 12.9 AST 5,080	5,835	Leukoerythroblastic film, predominantly monoblasts	–	Autopsy: confluent necrosis with multiacinar collapse and widespread infiltration by myeloid leukaemic cells	US: splenomegaly; normal liver with no biliary dilatation	Death
Eisen *et al.* [19]	74/M	WBC 8.76 (N 79%, L 6.5%, Mono 12%) Hgb 14.7 Plt 138	Tbil 8.4 Dbil 6.5 AST 859 ALT 443	3,357	No blasts	Infiltration by myeloid blast cells, positive to myeloperoxidase and vimentin	Autopsy: enlarged liver with multiple white and red nodules and numerous cystic- like areas and necrosis	CT: hypodense areas in the liver	Death
Our case	75/F	WBC 6.35 (N 63.3%, L 20.5%, M 13.4%) Hgb 8.9 Plt 30	Tbil 4.5 Dbil 2.4 AST 62 ALT 104		Rare blasts	Increased cellularity, diffuse infiltration by neoplastic cells and complete loss of myeloid maturation	Autopsy: diffuse infiltration of the liver by neoplastic cells and loss of normal structures	MRI: hepatomegaly and moderate splenomegaly; scattered nodular or mass-like areas of decreased T2-weighted signal within the hepatic and splenic parenchyma	Death

All laboratory values are of first encounters. Abbreviations and units: M, male; F, female; WBC, white blood cell X 109/L; Hgb, haemoglobin g/dl; Plt, platelets X 10^9^/L; N, neutrophils; L, lymphocytes; E, eosinophils; B, basophils; Tbil, total bilirubin in mg/dL; AST, aspartate aminotransferase U/L; DBil, direct bilirubin in mg/dL; ALT, alanine aminotransferase U/L; LDH, lactate dehydrogenase U/L; US, ultrasound; CT, computed tomography; MRI, magnetic resonance imaging; CBD, common bile duct
